# Author's reply

**Published:** 2010

**Authors:** Vinay B Agrawal

**Affiliations:** Clear Vision Eye Center, 1A, Ashoka, 15, S. V. Road, Santacruz (W), Mumbai-400 054, India

Dear Editor,

At the outset I thank Maskatifor the interest in the article.[1]

Point-wise clarification of the queries is given below.

- I had provided these figures so that the data presented to readers is complete.
- “All eyes completed was 12 months of follow up and 37 eyes had one year follow up” should read as “All 41 eyes completed the follow-up of six months, however, only 37 eyes were analyzed as they had completed the follow-up of one year”
- I apologize for the typographical errors as the final responsibility rests with me.
- For non-mention of the brand name against Propracaine, I am sure the readers will appreciate that there is no intent to “not publicize” the local brands. It is oversight for which I again am responsible.

I hope this allows the matter to rest.
